# Artificial intelligence algorithm for predicting cardiac arrest using electrocardiography

**DOI:** 10.1186/s13049-020-00791-0

**Published:** 2020-10-06

**Authors:** Joon-myoung Kwon, Kyung-Hee Kim, Ki-Hyun Jeon, Soo Youn Lee, Jinsik Park, Byung-Hee Oh

**Affiliations:** 1 Department of Critical Care and Emergency Medicine, Mediplex Sejong Hospital, 20, Gyeyangmunhwa-ro, Gyeyang-gu, Incheon, Republic of Korea; 2 Artificial Intelligence and Big Data Research Center, Sejong Medical Research Institute, Bucheon, South Korea; 3 Medical research team, Medical AI, co., Seoul, South Korea; 4 Medical R&D Team, Body Friend, co., Seoul, South Korea; 5 Division of Cardiology, Cardiovascular Center, Mediplex Sejong Hospital, Incheon, South Korea

**Keywords:** Heart arrest, Deep learning, Electrocardiography, Artificial intelligence, Hospital rapid response team

## Abstract

**Background:**

In-hospital cardiac arrest is a major burden in health care. Although several track-and-trigger systems are used to predict cardiac arrest, they often have unsatisfactory performances. We hypothesized that a deep-learning-based artificial intelligence algorithm (DLA) could effectively predict cardiac arrest using electrocardiography (ECG). We developed and validated a DLA for predicting cardiac arrest using ECG.

**Methods:**

We conducted a retrospective study that included 47,505 ECGs of 25,672 adult patients admitted to two hospitals, who underwent at least one ECG from October 2016 to September 2019. The endpoint was occurrence of cardiac arrest within 24 h from ECG. Using subgroup analyses in patients who were initially classified as non-event, we confirmed the delayed occurrence of cardiac arrest and unexpected intensive care unit transfer over 14 days.

**Results:**

We used 32,294 ECGs of 10,461 patients and 4483 ECGs of 4483 patients from a hospital were used as development and internal validation data, respectively. Additionally, 10,728 ECGs of 10,728 patients from another hospital were used as external validation data, which confirmed the robustness of the developed DLA. During internal and external validation, the areas under the receiver operating characteristic curves of the DLA in predicting cardiac arrest within 24 h were 0.913 and 0.948, respectively. The high risk group of the DLA showed a significantly higher hazard for delayed cardiac arrest (5.74% vs. 0.33%, *P* < 0.001) and unexpected intensive care unit transfer (4.23% vs. 0.82%, *P* < 0.001). A sensitivity map of the DLA displayed the ECG regions used to predict cardiac arrest, with the DLA focused most on the QRS complex.

**Conclusions:**

Our DLA successfully predicted cardiac arrest using diverse formats of ECG. The results indicate that cardiac arrest could be screened and predicted not only with a conventional 12-lead ECG, but also with a single-lead ECG using a wearable device that employs our DLA.

## Introduction

Cardiac arrest is a major public health burden and a recent study of in-hospital cardiac arrests in the United States estimates that 292,000 adults suffer cardiac arrest each year [1–3]. The study shows a concerning trend of cardiac arrest in approximately 38% greater than previously data [2, 4]. Although the survival rate of cardiac arrest has been increasing over the last two decades, the survival to hospital discharge was only 25% [5]. As up to 80% of patients show signs of deterioration before cardiac arrest, diverse rapid response systems (RRSs) have been implemented to prevent cardiac arrest in the past [6–8].

Several track and trigger systems (TTSs) using discrete numeric values such as vital signs and laboratory results are used in RRSs [9, 10]. As conventional TTSs have limitations in detecting deterioration in patients, several researchers have adopted deep learning based algorithms to deal with these numeric values, which performed better than conventional tools [11–15]. However, the performances of these novel TTSs were also not satisfactory, and further improvement is needed to use the algorithms with electrical health records. A paradigm shift is needed to use a new type of variable to improve the performance of predicting cardiac arrest.

Previous studies found QT prolongation, QRS prolongation, fragmented QRS complexes, and early repolarization to be associated with cardiac arrest [16–19]. However, it is not easy to detect such delicate changes in ECGs, and conventional statistical methods fail to build a criteria to predict cardiac arrest using the complex information of ECGs. The most important aspect of deep learning is its ability to extract features from high dimensional complex data and formulate algorithms from various types of data, such as images, two-dimensional (2D) data, and waveforms [20]. Recently, deep learning has been used to analyze ECGs for diagnosing left ventricular hypertrophy, aortic stenosis, atrial fibrillation, heart failure, and even determining age and sex [21–24]. We hypothesized that DLAs could effectively predict cardiac arrests. To test this hypothesis, we developed and validated a DLA for predicting cardiac arrest using ECGs.

## Methods

This study was approved by the institutional review boards (IRB) of Sejong general hospital (2018–0689) and Mediplex Sejong hospital (2018–054). Clinical data, including digitally stored ECGs, age, sex, and endpoints of admitted patients, were extracted from both hospitals. Both IRBs waived the need for informed consent because of the retrospective nature of the study, using fully anonymized ECG and health data, and minimal harm.

### ECG data

The predictor variables are ECG, age, and sex. Digitally stored 12-lead ECG data were recorded at 500 data points per second (500 Hz) at each lead for 10 s. We removed 1 s each at the beginning and end of the ECG, because these areas have more artifacts than other parts. Because of this, the length of each ECG was reduced to 8 s (4000). We made a dataset using the entire 12-lead ECG data. We also used partial datasets from the 12-lead ECG data, such as limb 6-lead (aVL, I, −aVR, II, aVF, and III) and single lead (I or II). We selected these leads as they can easily be recorded by wearable and pad devices in contact with the patient’s limbs [25]. Consequently, when we developed and validated an algorithm using 12-lead ECGs, we used the dataset that was 2D data of 12 × 4000 numbers. Similarly, for 6-lead and single lead ECGs, we used datasets comprising 6 × 4000 and 1 × 4000 numbers, respectively. We rearranged the input 2D ECG data in the order V1, V2, V3, V4, V6, aVL, I, −aVR, II, aVF, and III. Convolutional neural network (CNN) is a well-known deep learning architecture for learning 2D image data [20].

### Development of deep learning based artificial intelligence algorithm

**Fig. 1:**
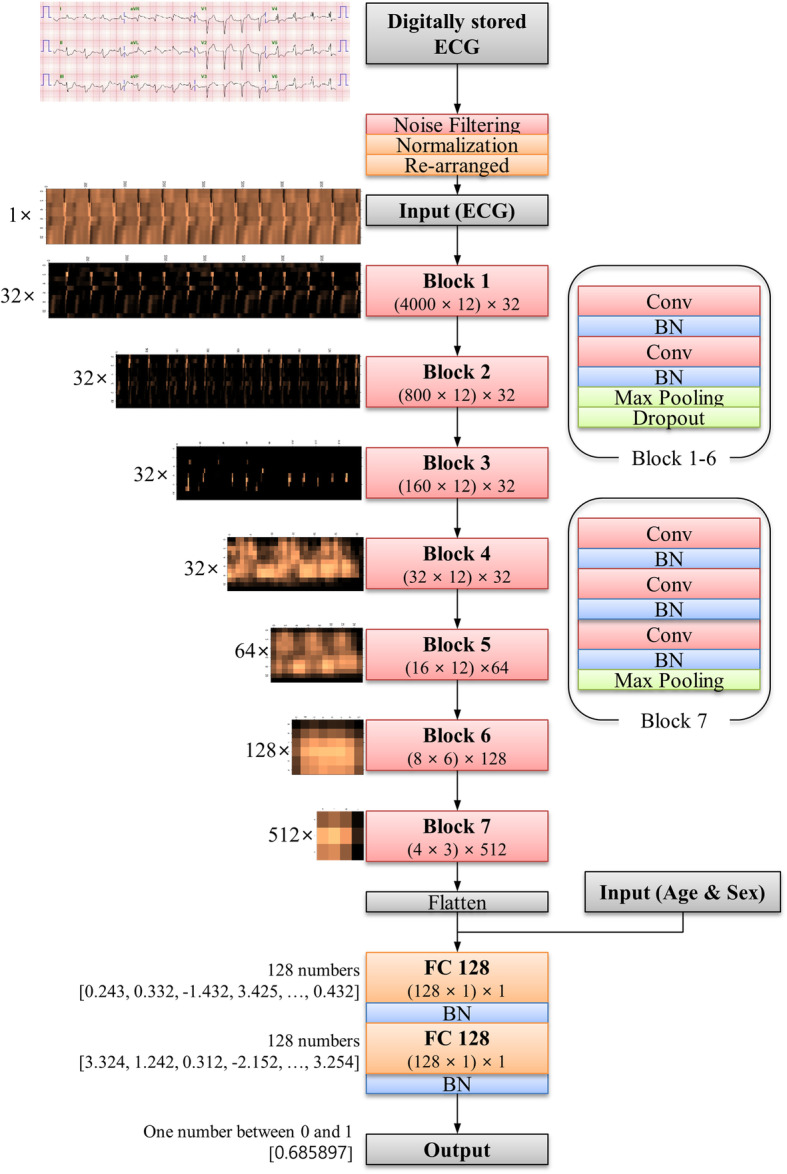
Architecture of deep-learning-based algorithm for predicting cardiac arrest. BN denotes batch normalization, Conv convolutional layer, ECG electrocardiography, and FC fully connected layer

The DLA was made using many hidden layers of neurons to learn complex hierarchical nonlinear representations from the data [20]. As a block with six stages, it had two convolutional layers, two batch normalization layers, one max pooling layer, and one dropout layer. This block was fully connected to the one-dimensional (1D) layer composed of 128 nodes (Fig. 1). The input layer of epidemiology (age, sex) was concatenated with the 1D layer. There were two fully connected 1D layers after the flattened layer, and the second layer was connected to the output node, which was composed of one node. The values of the output node represent the possibility of developing cardiac arrest, and the output node uses a sigmoid function as an activation function, as the output of the sigmoid function is between 0 and 1. We used TensorFlow’s open-source software library (Google LLC, Mountain View, CA USA) as the backend, and conducted our experiment with Python (version 3.5.2; Python Software Foundation, Beaverton, OR, USA). We conducted additional experiments for the DLA using limb 6-lead and each single-lead (lead I, lead II, lead III, aVR, aVL, and V1–6) ECGs. To develop and validate the DLA for these ECGs, we changed the sizes of the filters and convolutional layers, thus adjusting the shape of the input datasets. The number of filters, max pooling, and fully connected layers were the same as that of the 12-lead ECG architecture.

### Development and validation datasets

**Fig. 2:**
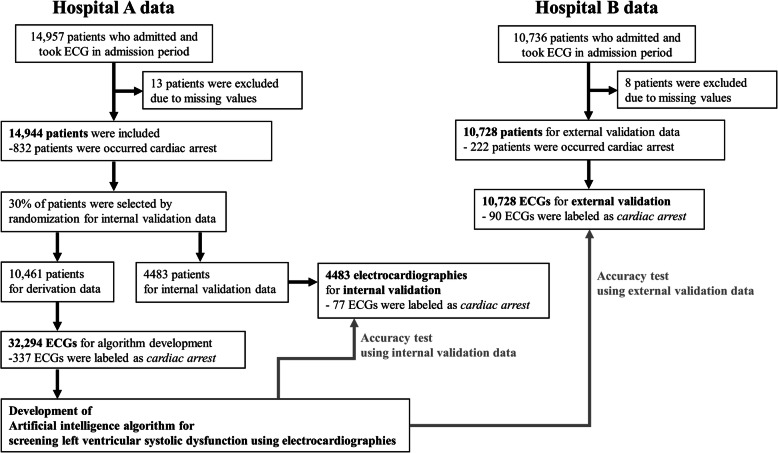
Study flowchart

Data from hospital A were used for development and internal validation. We identified patients who were admitted to hospital A in the study period (October 2016–September 2019), and who had at least one standard digital, 10 s, 12-lead ECG acquired in the supine position during the admission period. We excluded subjects with missing demographic or electrocardiographic information. As shown in Fig. 2, patients treated at hospital A were randomly and exclusively split into algorithm development (70%) and internal validation (30%) datasets. Data from hospital B were only used for external validation, which confirmed that the developed DLA was robust across diverse datasets. The characteristics of the 2 hospitals are different (hospital A is a cardiovascular teaching hospital, and hospital B is a community general hospital). We also identified patients who were admitted to hospital B in the study period (March 2017–September 2019) and had at least one ECG during it. We also excluded subjects in hospital B with missing values. Because the purpose of the validation data was to assess the accuracy of the algorithm, we used only one ECG from each patient for the internal and external validation dataset—the most recent ECG to the endpoints (cardiac arrest or survival and subsequent discharge).

### Endpoint

The endpoint of this research was cardiac arrest, defined as a lack of palpable pulse, with or without attempted resuscitation. We reviewed electronic health records to identify the exact time of each endpoint. The objective of the DLA was to predict whether an ECG was within the prediction time window of cardiac arrest, which is the 24 h interval before cardiac arrest. For a patient with cardiac arrest, the ECGs belonging to the prediction window were labeled as *cardiac arrest* and other ECGs were labeled as a *nonevent*. For a patient without cardiac arrest, all ECGs were labeled as a *nonevent*. In other words, the aim of the developed DLA was to accurately classify an ECG as *cardiac arrest* or *nonevent*.

### Statistical analysis

At each input (ECG, age, and sex) of the validation data, the DLA calculated the possibility of cardiac arrest in the range from 0 (*nonevent*) to 1 (*cardiac arrest*). To confirm the performance of the DLA, we compared the possibility calculated by the DLA with the occurrence of cardiac arrest within 24 h after the time of ECG in the validation data. For this, we used the area under the receiver operating characteristics curve (AUROC) to measure the performance of the model. As the purpose of the DLA was screening, we evaluated the specificity, the positive predictive value, and the negative predictive value at a cut-off point selected for high (90%) sensitivity in development data. Exact 95% confidence intervals (CIs) were used for all measures of diagnostic performance except for AUROC. The CI for AUROC was determined based on Sun and Su optimization of the De-long method, using the pROC package in R (The R Foundation, Vienna, Austria; www.r-project.org). Statistical significance for the differences in patient characteristics was defined as a 2-sided *P* value of less than 0.001. Measures of the diagnostic performance were summarized using 2-sided 95% CIs. Analyses were computed using R software, version 3.4.2.

### Subgroup analysis

We hypothesized that early in the course of any deterioration, ECG signals would show subtle abnormal patterns due to metabolic and structural changes. Although cardiac arrest did not happened within 24 h in *nonevent* ECGs, delayed cardiac arrest and events of deterioration could have occurred in nonevent ECGs as well. In other words, we hypothesized that our DLA would classify ECGs with characteristics of deterioration as *cardiac arrest*, giving the initial appearance of a false positive test (that is, an ECG classified as *cardiac arrest*, but not leading to cardiac arrest within 24 h). To test this hypothesis, we designed two subgroup analyses with *nonevent* ECGs in the external validation dataset. We divided the *nonevent* ECGs as low and high risk groups defined by the DLA. In the analysis of the first subgroup, we confirmed the occurrence of cardiac arrest over 2 weeks in each ECG. We also confirmed the performance of the DLA in predicting deterioration events. The deterioration events were defined as unexpected intensive care unit transfer over 2 weeks in each ECG. In the *nonevent* ECGs of the external validation data, we included ECGs which were acquired in general wards for second subgroup analysis. Kaplan-Meier analysis was used to depict the occurrence of delayed cardiac arrest and deterioration events for the true negative (low risk) versus the false positive (high risk) groups over time. Subsequently, Cox proportional hazards regression was used to estimate the hazard for the delayed cardiac arrest and the deterioration events.

### Visualizing using sensitivity map

To understand the developed DLA and make a comparison with existing medical knowledge, it was important to identify which regions had significant effects on the decision of the DLA. We employed a sensitivity map using the saliency method, and used it to visualize the ECG regions used by the DLA to predict cardiac arrest. The map was computed using the first-order gradients of the classifier probabilities with respect to the input signals. If the probability of a classifier was sensitive to a specific region of the signal, the region would be considered as significant in the model. We used a gradient-weighted class activation map (Grad-CAM) for visualization [26]. Grad-CAM uses the gradient information of the algorithm, and could be used with any activation function and any architecture of CNNs.

## Results

The study population included 47,505 ECGs of 25,672 patients, in which cardiac arrest occurred in 1054 patients, as shown in Fig. 2. The number of ECGs from 1054 cardiac arrest patients was 2298. Of those 2298 ECGs, the number of ECGs labeled as *cardiac arrest* was 504. The development dataset from hospital A included 32,294 ECGs of 10,461 patients. The performance of the DLA was then confirmed using 4483 ECGs from the 4483 patients in the internal validation data from hospital A, and 10,728 ECGs from the 10,728 patients in the external validation data from hospital B. Baseline characteristics of study population were shown in Table 1.

**Table 1:** Baseline characteristics

Variables	Non-event patients ***n*** = 24,618	Cardiac arrest patients ***n*** = 1054	***P***-value
Male, n (%)	13,072 (53.1)	558 (52.9)	0.888
Age, year (mean (sd))	60.67 (16.69)	72.37 (13.24)	< 0.001
Admission to ICU, n (%)	279 (1.1)	638 (60.5)	< 0.001
Emergent admission, n (%)	10,491 (42.6)	800 (75.9)	< 0.001
Admission division, n (%)			< 0.001
Cardiovascular	13,836 (56.2)	760 (72.1)	
Cerebrovasclar	2474 (10.0)	142 (13.5)	
Respiratory disease	810 (3.3)	63 (6.0)	
Other internal medicines	2934 (11.9)	64 (6.1)	
Major surgery	4306 (17.5)	18 (1.7)	
Others	258 (1.0)	7 (0.7)	
Length of stay, day (mean (sd))	9.34 (15.20)	54.35 (114.36)	< 0.001
Heart rate, bpm (mean (sd))	74.57 (17.76)	93.43 (27.91)	< 0.001
PR interval, msec (mean (sd))	172.03 (31.72)	170.65 (41.28)	0.262
QT interval, msec (mean (sd))	406.41 (47.17)	393.70 (71.65)	< 0.001
QTc (mean (sd))	445.79 (38.02)	474.27 (46.81)	< 0.001
QRS duration, msec (mean (sd))	97.25 (19.25)	107.62 (28.58)	< 0.001
P wave axis (mean (sd))	43.20 (31.26)	44.89 (47.43)	0.190
R wave axis (mean (sd))	36.14 (46.52)	39.15 (75.26)	0.046
T wave axis (mean (sd))	51.17 (59.34)	86.39 (92.21)	< 0.001

**Fig. 3:**
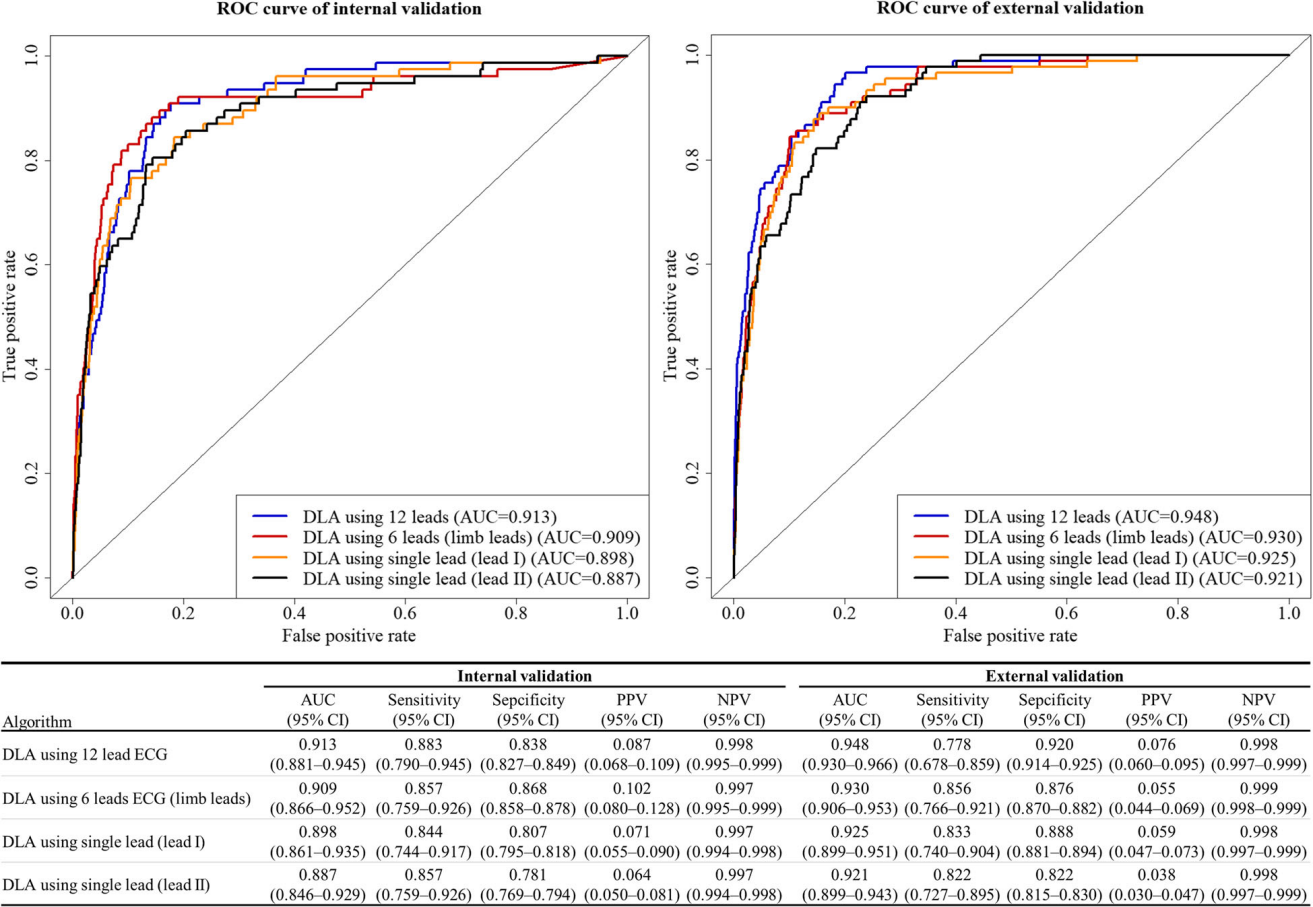
Performances of artificial intelligence algorithms for predicting cardiac arrest. AUC denotes area under the receiver operating characteristic curve, CI confidence interval, DLA deep-learning based artificial intelligence algorithm, NPV negative predictive value, PPV positive predictive value, and ROC receiver operating characteristic curve

For endpoint (*cardiac arrest* within 24 h of ECG), the AUROC of the 12-lead DLA were 0.913 (95% confidence interval: 0.881–0.945) and 0.948 (0.930–0.966) during internal and external validation, respectively. As shown in Fig. 3, the AUROC was 0.004 to 0.027 lower when using 6-lead or single-lead ECG than when using 12-lead ECG. As shown in the Supplemental material 1, when we confirmed the performance of DLA using a single lead, a DLA using lead I outperformed other DLAs using other leads.

**Fig. 4:**
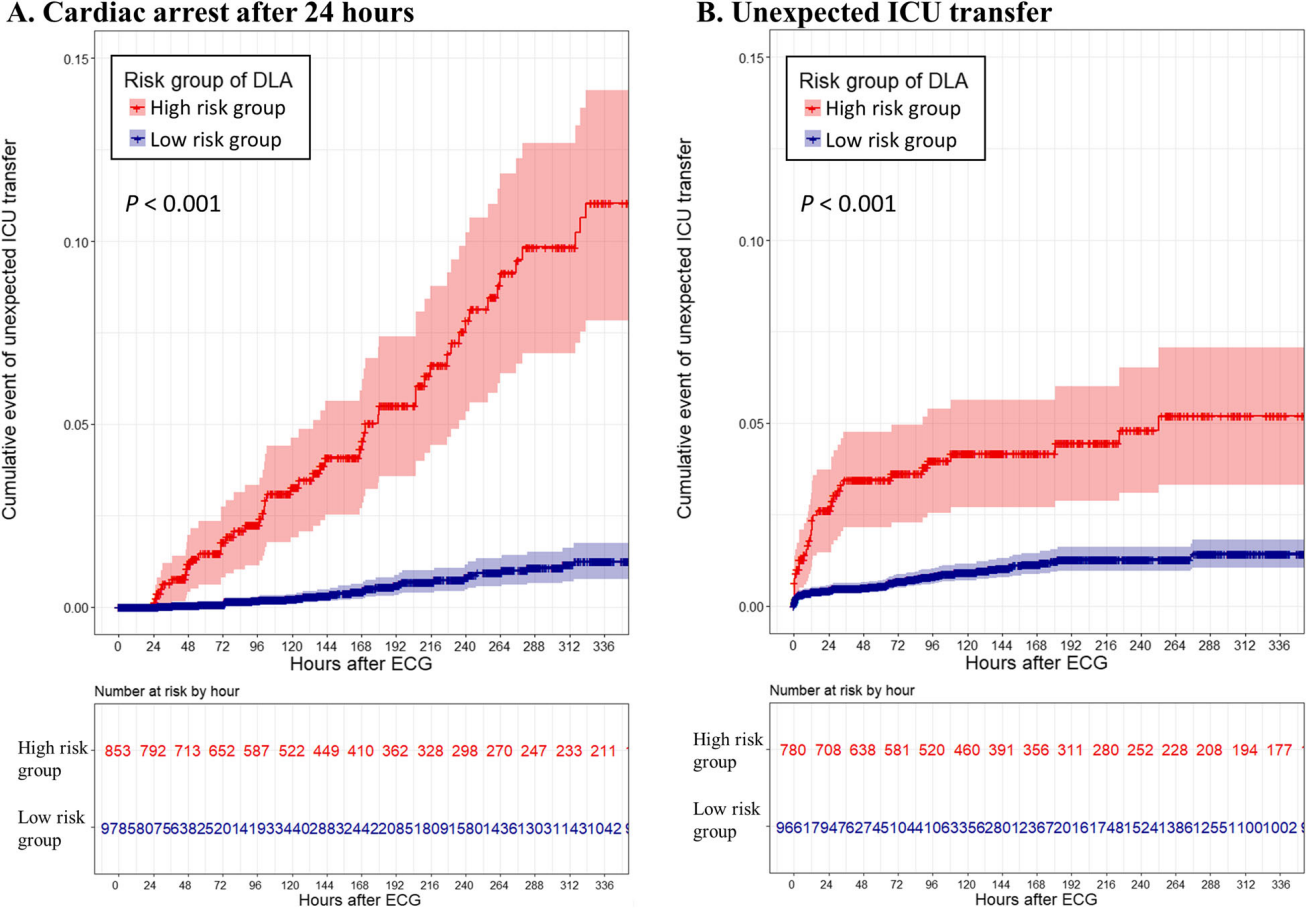
Cumulative hazard of deterioration event in patients who had no cardiac arrest within 24 h. DLA denotes deep-learning based artificial intelligence algorithm, ECG electrocardiography, and ICU intensive care unit. The cutoff point used for dividing the risk groups was selected when the overall sensitivity was 90% in the development dataset. Cox proportional hazards regression was used to estimate the hazard for the delayed cardiac arrest and the deterioration events

In the external validation data, there were 10,638 ECGs from 10,638 patients labeled as *nonevent*. We conducted first subgroup analysis of the cardiac arrest over 2 weeks with these 10,638 ECGs. Of them, cardiac arrest occurred within 2 weeks in 81 ECGs. The high risk group of the DLA showed a significantly higher hazard (Fig. 4) and higher occurrence rate of cardiac arrest than the low risk group (5.74% vs. 0.33%, *P* < 0.001). The second subgroup analysis was performed with 10,441 ECGs acquired in the general ward. Of them, unexpected intensive care unit transfer within 2 weeks occurred in 112 ECGs. The high risk group of the DLA showed a significantly higher hazard (Fig. 4) and higher occurrence rate of unexpected intensive care unit transfers than the low risk group (4.23% vs. 0.82%, *P* < 0.001).

**Fig. 5:**
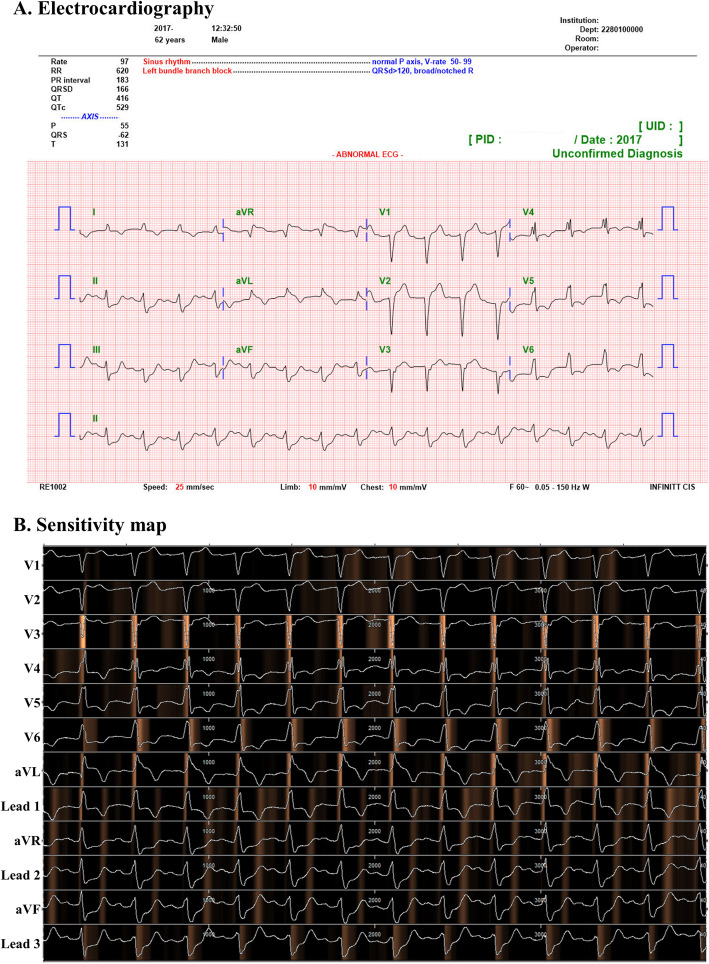
Electrocardiography and sensitivity map of patient with cardiac arrest. This is electrocardiography of patient who was 62 years old and was occurred cardiac arrest in external validation hospital. The cardiac arrest occurred 18 min after acquiring electrocardiography. The deep learning based artificial intelligence algorithm predicted cardiac arrest in this patient with a value of 0.685897, which was 32.7 times the cut-off value of sensitivity 90% in development dataset

As shown in Fig. 5, the sensitivity map shows that the DLA focused mostly on the QRS complex to predict cardiac arrest. The DLA also focused on T-wave, but the significances of these regions were lower than that of the QRS complex.

## Discussion

This is the first study that developed and validated a DLA for predicting cardiac arrest using ECGs. This study reveals that a deep-learning algorithm, one of the powerful tools of artificial intelligence, can figure out very delicate ECG changes in predicting cardiac arrest. The performance for predicting cardiac arrest was preserved with 6-lead or single-lead DLAs. In recent years, there many wearable devices for monitoring ECGs have been developed [27]. If cardiac arrest could be predicted using ECGs, one can in principle capture the risk of patients in general wards or at home through wearable devices. Additionally, ECGs as raw bio-signal data can be used, which can enhance the performance of recent TTSs based on deep learning.

In development and validation using retrospective data of more than 46,000 ECGs, the DLA had a high AUROC of 0.913 to 0.948 for predicting cardiac arrest. The model showed good performance using data from another hospital that was not used for algorithm development, and had different patient characteristics and data shape. At a high-sensitivity (90%) operating point in development data, the DLA performed well as a potential TTS to predict cardiac arrest, and screen risk in patients with a negative predictive value greater than 99.8%. The model’s performance was better than conventional TTSs, and similar to recent novel TTSs based on deep learning. Also, the model’s performance was better than other commonly used screening tests such as mammography for breast cancer (AUROC, 0.78, positive predictive value, 3–12%), and fecal occult blood testing for detecting colorectal neoplasia (AUROC 0.71, overall sensitivity, 29%) [28, 29].

The most important aspect of deep learning is its ability to extract features and make an algorithm from various types of data, such as images, 2D data, and waveforms. Here, we used raw ECG data (2D numerical data, 12 **×** 4000) and interpreted ECG patterns for predicting cardiac arrest. Attia et al. developed deep-learning algorithms for screening cardiac contractile dysfunction, predicting the occurrence of atrial fibrillation during sinus rhythm, approximating age and sex, and detecting hyperkalemia using raw ECG data and demonstrated its feasibility.^11,12^ Our study group showed that a deep-learning-based algorithm using ECG could outperform cardiologists in diagnosing left ventricular hypertrophy and diagnosis aortic stenosis.^13^ However, deep learning is often criticized for the unreliability of its outcomes because of the unpredictability of the process. Because of this, we used a sensitivity map to visualize the regions of the ECGs that were used for decision-making by the DLA.

The map shows that the DLA focused more on the QRS complex to decide and predict cardiac arrest. The DLA also partially focused on the T-wave for predicting cardiac arrest. QRS prolongation has been considered a prognostic marker for mortality among patients with a variety of cardiovascular diseases [16–18]. Moreover, QRS fragmentation has been reported to be associated with increased mortality in patients with structural heart disease [19]. In this study, the *cardiac arrest* ECGs had prolonged QRS durations and QTs corrected. The heart rates of *cardiac arrest* ECGs were greater than that of *nonevent* ECGs, and the T wave axes of *cardiac arrest* ECGs were more rightward than that of *nonevent* ECGs. As shown in the Supplemental material 2, we described the features of the high- and low-risk ECG of the DLA. The high-risk ECGs had tachycardia, wide QRS duration. However, because of the limitations of deep learning, we could not determine the exact process of calculation of the risk score by the DLA. Conducting research of explainable deep learning technologies for ECG will be the focus of our next study.

We described the features of cardiac arrest patients in the validation dataset in a supplemental material. As shown in the Supplemental material 3, the DLA could predict cardiac arrest in several young patients. Although a prospective study is needed to prove the clinical improvement, it is possible to improve clinical outcomes using the DLA in the general ward.

Although the developed DLA could detect the deterioration in a patient, several factors impede its application in real clinical practice. Owing to the lack of required resources in the ICU, we could not transfer all high-risk patients to the ICU. However, we could use tele-monitoring devices to monitor high-risk patients, enabling the physicians of the rapid response team to reevaluate these patients. Moreover, it is important to note that it is difficult to prove clinical improvement; if cardiac arrests were prevented due to increased monitoring of patients, this outcome would not be measurable. Therefore, previous studies on alarm detection systems for deterioration in patients have focused on the transfer of patients to the ICU as the endpoint.

Our study has several limitations to be resolved in the future. First, this was a retrospective study using conventional 12-lead ECGs. A prospective study is warranted to determine the association of the DLA, and enhancement in detecting cardiac arrest and improving clinical outcomes. In such future studies, it is important that we exclude patients who experienced cardiac arrest without any resuscitation attempts because such supposed clinical improvements possess little perceived value, especially if the patients are not resuscitated. A study for confirming the accuracy of data from various wearable or portable ECG devices is warranted to apply the DLA to those devices. If we adopt DLAs in daily living, a study is also needed to confirm the performance at home and general environments. Second, the performance of the DLA needs to be enhanced in order to use it as a reliable cardiac arrest detecting tool. Although the NPV was over 99%, the PPV was only 8% at the point of high sensitivity. As there are several methodologies that have been developed in deep learning and computer science, we could develop higher performance DLAs in the near future. We also developed a high performance TTS, by combining the DLA with discrete numeric variables such as the vital sign. Thirdly, we need to explore the decision-making process of the DLA further. For example, additional experiments are required to understand the deep learning process better, and thereby understand which exact characteristics of the QRS complex and the T wave influence the algorithm’s decision. Explainable artificial intelligence has been studied and reported on recently, so the “black box” limitation could be solved in the near future [30]. This subject will be our next area of study, and this might turn out to be the new standard for discovering new medical knowledge about diseases and ECGs.

## Conclusion

The newly developed deep learning-based artificial intelligence algorithm demonstrated a high performance in predicting cardiac arrest using not only a 12-lead, but also a single-lead ECG. The results indicate that cardiac arrest could be screened and predicted not only with a conventional 12-lead ECG, but also with a single-lead ECG using a wearable device that employs the artificial intelligence algorithm.

## Supplementary information

**Additional file 1: Supplemental material 1.** Performance of deep learning algorithm based on single lead electrocardiography.

**Additional file 2: Supplemental material 2.** Electrocardiographic features of high and low risk group defined by deep learning based algorithm.

**Additional file 3: Supplemental material 3.** Features of cardiac arrest patients in validation datasets.

## Data Availability

The datasets used and/or analyzed during the current study are available from the corresponding author on reasonable request.

## Abbreviations

AUROC: Area under the receiver operating characteristics curve
CI: Confidence interval
CNN: Convolutional neural network
DLA: Deep-learning-based artificial intelligence algorithm
ECG: Electrocardiography
Grad-CAM: Gradient-weighted class activation map
IRB: Institutional review board
RRS: Rapid response system
TTS: Track and trigger system
1D: One-dimensional
2D: Two-dimensional
