# Supplementary material for: Artificial intelligence algorithm for predicting cardiac arrest using electrocardiography
Source: Scand J Trauma Resusc Emerg Med. 2020 Oct 6;28:98. doi: 10.1186/s13049-020-00791-0 (PMC7541213; doi:10.1186/s13049-020-00791-0)
Supplement: Supplementary file 1 — Additional file 1: Supplemental material 1. Performance of deep learning algorithm based on single lead electrocardiography. [file 13049_2020_791_MOESM1_ESM.docx]

**Supplemental material 1 Performance of deep learning algorithm based on single lead electrocardiography**

|  |  | **Internal validation AUC (95% CI)** | **External validation AUC (95% CI)** |
| --- | --- | --- | --- |
| **Limb lead** | **I** | 0.898 (0.861–0.935) | 0.925 (0.899–0.951) |
|  | **II** | 0.887 (0.846–0.929) | 0.921 (0.899–0.943) |
|  | **III** | 0.871 (0.843–0.914) | 0.902 (0.881–0.919) |
|  | **aVL** | 0.875 (0.851–0.908) | 0.912 (0.890–0.925) |
|  | **aVR** | 0.881 (0.861–0.901) | 0.915 (0.892–0.929) |
|  | **aVF** | 0.869 (0.838–0.899) | 0.900 (0.879–0.915) |
| **Precordial Lead** | **V1** | 0.884 (0.853–0.911) | 0.911 (0.894–0.927) |
|  | **V2** | 0.879 (0.849–0.903) | 0.908 (0.887–0.925) |
|  | **V3** | 0.873 (0.842–0.891) | 0.901(0.878–0.914) |
|  | **V4** | 0.870 (0.841–0.898) | 0.899 (0.875–0.911) |
|  | **V5** | 0.887 (0.864–0.912) | 0.916 (0.891–0.935) |
|  | **V6** | 0.891 (0.858–0.924) | 0.920 (0.893–0.941) |

AUC denotes area under the receiver operating characteristic curve and CI confidence interval.
