# Supplementary material for: Artificial intelligence algorithm for predicting cardiac arrest using electrocardiography
Source: Scand J Trauma Resusc Emerg Med. 2020 Oct 6;28:98. doi: 10.1186/s13049-020-00791-0 (PMC7541213; doi:10.1186/s13049-020-00791-0)
Supplement: Supplementary file 2 — Additional file 2: Supplemental material 2. Electrocardiographic features of high and low risk group defined by deep learning based algorithm. [file 13049_2020_791_MOESM2_ESM.docx]

**Supplemental material 2 Electrocardiographic features of high and low risk group defined by deep learning based algorithm**

|  | **Low risk ECG  of DLA** | **High risk ECG  of DLA** | ***p*** |
| --- | --- | --- | --- |
| **Male, %** | 60.9 | 51.6 | <0.001 |
| **Age, year (mean (SD))** | 57.27 (15.02) | 73.71 (12.24) | <0.001 |
| **Heart rate, bpm (mean (SD))** | 60.44 (5.49) | 106.95 (26.98) | <0.001 |
| **PR interval, msec (mean (SD))** | 175.13 (28.53) | 164.69 (48.14) | <0.001 |
| **QRS duration, msec (mean (SD))** | 93.38 (11.34) | 110.90 (31.19) | <0.001 |
| **QT interval, msec (mean (SD))** | 426.31 (32.67) | 370.32 (65.14) | <0.001 |
| **QTc (mean (SD))** | 426.64 (29.56) | 480.99 (52.93) | <0.001 |
| **P wave axis (mean (SD))** | 41.68 (25.46) | 46.49 (48.93) | <0.001 |
| **R wave axis (mean (SD))** | 41.68 (32.36) | 33.09 (71.16) | <0.001 |
| **T wave axis (mean (SD))** | 48.08 (47.12) | 105.44 (90.42) | <0.001 |

DLA denotes deep-learning-based artificial intelligence algorithm
