# Supplementary material for: Artificial intelligence algorithm for predicting cardiac arrest using electrocardiography
Source: Scand J Trauma Resusc Emerg Med. 2020 Oct 6;28:98. doi: 10.1186/s13049-020-00791-0 (PMC7541213; doi:10.1186/s13049-020-00791-0)
Supplement: Supplementary file 3 — Additional file 3: Supplemental material 3. Features of cardiac arrest patients in validation datasets. [file 13049_2020_791_MOESM3_ESM.docx]

**Supplemental material 3 Features of cardiac arrest patients in validation datasets**

|  | **Low risk group of DLA (n=31)** | **High risk group of DLA (n=136)** | **p** |
| --- | --- | --- | --- |
| **Male, n (%)** | 15 ( 48.4) | 79 ( 58.1) | 0.434 |
| **Age group, n (%)** |  |  | 0.338 |
| **<40** | 1 ( 3.2) | 1 ( 0.7) |  |
| **40 - 50** | 4 ( 12.9) | 7 ( 5.1) |  |
| **50 - 60** | 5 ( 16.1) | 16 ( 11.8) |  |
| **60 - 70** | 3 ( 9.7) | 27 ( 19.9) |  |
| **70 - 80** | 17 ( 54.8) | 82 ( 60.3) |  |
| **>90** | 1 ( 3.2) | 3 ( 2.2) |  |
| **Heart rate, bpm (mean (SD))** | 78.39 (25.16) | 104.45 (29.45) | <0.001 |
| **death24 = 1 (%)** | 14 ( 45.2) | 91 ( 66.9) | 0.04 |
| **Cardiac arrest with  resuscitation, n (%)** | 23 ( 74.2) | 75 ( 55.1) | 0.082 |

DLA denotes deep-learning-based artificial intelligence algorithm
